# Bioresorbable Scaffolds: Current Technology and Future Perspectives

**DOI:** 10.5041/RMMJ.10402

**Published:** 2020-04-29

**Authors:** Brian Forrestal, Brian C. Case, Charan Yerasi, Anees Musallam, Chava Chezar-Azerrad, Ron Waksman

**Affiliations:** Section of Interventional Cardiology, MedStar Washington Hospital Center, Washington, DC, USA

**Keywords:** Bioresorbable scaffold, drug-eluting stent, stent thrombosis, target lesion failure, target lesion revascularization

## Abstract

Metallic drug-eluting stents have led to significant improvements in clinical outcomes but are inherently limited by their caging of the vessel wall. Fully bioresorbable scaffolds (BRS) have emerged in an effort to overcome these limitations, allowing a “leave nothing behind” approach. Although theoretically appealing, the initial experience with BRS technology was limited by increased rates of scaffold thrombosis compared with contemporary stents. This review gives a broad outline of the current BRS technologies and outlines the refinements in BRS design, procedural approach, lesion selection, and post-procedural care that resulted from early BRS trials.

## INTRODUCTION

Since their introduction in the mid-1980s, coronary stents have evolved significantly and have led to improvements in the treatment of patients with coronary artery disease. The prevention of acute and subacute vessel closure due to vessel recoil by the initial bare metal stents led to reductions in restenosis rates, a major limitation of balloon angioplasty.^1^ Subsequent generations of stents, with the addition of antiproliferative agents and polymer coatings, led to even further improvements in restenosis rates, and to the widespread adoption of the technology.^2^

As with any new technology, however, there were limitations with leaving a permanent scaffold within the vessel. These included impaired normal vasomotor reactivity^3^ and jailing of side branches. Other concerns included initiating the inflammatory host response to the polymer coatings of the device at the vessel wall, leading to neointimal hyperplasia, restenosis, and stent thrombosis.^4^ Bioresorbable scaffolds (BRS) were developed to provide all of the short-term benefits of permanent stents but with the added benefit of completely degrading over the medium- to long-term period, allowing full recovery of vasomotor and endothelial function.^5^,^6^ This strategy of “leave nothing behind” aimed to prevent long-term inflammation, preserve distal bypass grafting sites, and allow unimpeded future vessel imaging.

Although the theoretical benefits of BRS were attractive, the initial generation of BRS devices, principally the Absorb Bioresorbable Vascular Scaffold (Abbott Vascular, Santa Clara, CA, USA), were hampered by increased rates of stent thrombosis compared with contemporary stents.^7^,^8^ This led to the withdrawal of the product from the market in 2017, and a loss of trust in the technology. Newer-generation devices utilizing different bioresorbable materials and featuring improved stent strut thickness may still provide a viable path to the “leave nothing behind” strategy.

Here, we aim to provide an outline of the development of the current BRS technologies and an overview of the field’s future directions.

## CURRENT DEVICES

The majority of BRS that have been brought to the clinical trial stage have been based on lactate polymers. Other materials utilized include magnesium alloys, tyrosine copolymers, and iron. Table 1 out-lines some of the major BRS that have been brought to clinical trials. The following section outlines some of the key materials used in BRS and design features of the representative devices, with Table 2 outlining their clinical performance. The technologies used for BRS can be broadly categorized as polymeric resorbable scaffolds or metallic resorbable scaffolds (MRS).

**Table 1:** Key Design Characteristics of Current Bioresorbable Scaffold Technologies.

Device (Manufacturer)	Drug	Backbone Material	Strut Thickness (μm)	Bioresorption Time (months)	EU CE Mark	FDA Approval
ABSORB GT1 BRS (Abbott)	Everolimus	PLLA	156	24–36	Jan 2011	July 2016*
DESolve (Elixir Medical)	Novalimus	PLLA	150	24–36	May 2014	No
ART Pure (Arterial Remodelling Technologies)	Drug-free	PDLLA	170	12–24	May 2015	No
MeRes 100 (Meril Life Sciences)	Sirolimus	PLLA	180	24	August 2019	No
FORTITUDE (Amaranth Medical)	Sirolimus	PLLA	150	12–24	No	No
APTITUDE (Amaranth Medical)	Sirolimus	PLLA	115	12–24	No	No
MAGNITUDE (Amaranth Medical)	Sirolimus	PLLA	98	12–24	No	No
DEFIANCE (Amaranth Medical)	Sirolimus	PLLA	85	12–24	No	No
Mirage (Manli)	Sirolimus	PLLA	125–150	14	No	No
NeoVas (Lepu Medical Technology)	Sirolimus	PLLA	180	36	No	No
Firesorb (Shanghai MicroPort)	Sirolimus	PLLA	100–125	36	No	No
Falcon (Abbott)	Everolimus	PLLA	<100		No	No
Fantom (REVA Medical)	Sirolimus	DAT	125	12	April 2017	No
Magmaris (Biotronik)	Sirolimus	Magnesium	120–150	12	June 2016	No
IBS (Lifetech Scientific)	Sirolimus	Iron	70	>12	No	No

* US sales discontinued September 2017.

DAT, Desaminotyrosine polycarbonate; PDLLA, poly-D, L-lactic acid; PLLA, poly-L-lactic acid.

Modified with updated data from Jinnouchi H et al.^6^ with permission, ©2018 Springer Nature.

**Table 2:** Key Clinical Performance Measures of Selected Bioresorbable Scaffolds.

Device (Manufacturer)	Patients Enrolled	Angiographic Follow-up (months post implant)	Late Lumen Loss (mm)	Clinical Follow-up Period (months)	TLF (%)	Scaffold Thrombosis (%)	Ischemia-driven TLR (%)
ABSORB GT1 BRS (Abbott)	2161*	6	0.19±0.18	60	11.6	2.5	8.4
DESolve Nx (Elixir Medical)	122	6	0.20±0.32	60	7.4	0	4.1
ART Pure (Arterial Remodelling Technologies)	30	6	-	-	-	-	-
MeRes 100 (Meril Life Sciences)	108	6	0.15±0.23	12	-	0	0.9
FORTITUDE (Amaranth Medical)	63	24	0.27±0.37	24	4.9	1.8	5.3
APTITUDE (Amaranth Medical)	60	9	0.33±0.36	24	3.4	0	0
MAGNITUDE (Amaranth Medical)	70	9	0.19±0.16	9	2.9	0	0
Mirage (Manli)	35	12	0.37±0.14	12	17.2	3.4	17.2
NeoVas (Lepu Medical Technology)	1103	MSCT follow-up	-	12	3.0	0.5	1.7
Fantom (REVA Medical)	117	6	0.25±0.40	24	4.2	0.8	2.9
Magmaris (Biotronik)	1075	12	0.52±0.39	36	4.3	0.5	2.4

* Pooled analysis of ABSORB II, ABSORB JAPAN, ABSORB CHINA, ABSORB III; >150,000 commercially treated patients worldwide.

MSCT, multi-slice computed tomography; TLF, target lesion failure; TLR, target lesion revascularization.

Modified with updated data from Jinnouchi H et al.^6^ with permission, ©2018 Springer Nature.

### Poly-lactate-based BRS

The majority of the data on BRS has been provided by lactate-based polymer systems, with poly-L-lactic acid (PLLA) the most commonly used polymer. The PLLA polymer is a thermoplastic aliphatic polyester that undergoes hydrolysis upon contact with the blood pool into lactate monomers and, ultimately, water and carbon dioxide when metabolized by the Krebs cycle.^9^,^10^ For many years PLLA has been used in a variety of other applications, such as resorbable sutures. Compared with metal alloys such as cobalt chromium and stainless steel, which are typically used in modern stents, PLLA has a lower tensile strength and, therefore, requires significantly thicker struts to provide comparable radial strength.^11^ Poly-D, L-lactic acid (PDLLA) undergoes a similar breakdown process to PLLA but at a faster rate because of a decreased crystalline structure compared to PLLA.

Several PLLA and PDLLA BRS exist at various stages of development, but the ABSORB clinical program has provided the majority of data on lactate-based BRS.

#### The Absorb BRS program

The Absorb BRS was the first BRS approved for use in the United States by the US Food and Drug Administration on the basis of several large, multicenter, randomized controlled trials. The ABSORB II, ABSORB III, ABSORB CHINA, ABSORB JAPAN, TROFI II, and EVERBIO III trials^8^,^12^–^16^ were all large-scale, multicenter, prospective trials comparing Absorb to a contemporary cobalt chromium everolimus-eluting stent (CoCr-EES) (Abbott Vascular, Santa Clara, CA, USA). Although individual trial-level results demonstrated similar performance characteristics and safety profiles between the two stents, subsequent analyses demonstrated increased rates of scaffold thrombosis at 1 year in the Absorb cohort.^17^,^18^ This trend appeared to continue to 3 years,^19^ with the additional worrisome finding of increased ischemia-driven target lesion revascularization (TLR) in the Absorb group.^7^ Longer-term follow-up to 4 and 5 years post-implantation demonstrated that the risk of adverse events appeared to stabilize after 3 years and was comparable to CoCr-EES,^12^ likely reflecting the complete resorption of the scaffold within the vessel wall.

The short- to medium-term concerns raised by the ABSORB program led to the withdrawal from the US market in 2018.^4^ However, the program provided some valuable lessons, in particular in BRS deployment techniques. Optimal deployment techniques that aimed at reducing inadequate vessel sizing, malposition, and scaffold underexpansion appeared, in smaller sub-studies, to reduce the rates of scaffold thrombosis, and the “PSP” technique (pre-dilation, proper sizing, and post-dilation) emerged. These findings were not consistent across all studies examining the effect of pre-dilation and post-dilation, and it remains unclear whether the risk of scaffold thrombosis related to the Absorb device or the deployment technique.^20^

### Desaminotyrosine Polycarbonate-based BRS

Desaminotyrosine polycarbonate (DAT) is a polycarbonate copolymer of tyrosine analogues and is combined with biocompatible hydroxyesters when used in BRS. The DAT polymer has similar radial strength and recoil characteristics to metallic stents^21^ and has the added benefit of allowing combination with low levels (3%) of iodine to allow improved visualization under fluoroscopy.^6^,^21^

#### The FANTOM program

The Fantom stent (REVA Medical, San Diego, CA, USA), based on DAT, has a 125-μm strut and incorporates iodine into the scaffold to improve visualization. Upon breakdown, the stent elutes sirolimus, with 80% of strut degradation occurring in the first 12 months and complete resorption occurring at 36 months.^21^ Additionally, the strut design and DAT allow for single inflation.

The FANTOM II study enrolled 240 patients across 28 sites and demonstrated promising safety and efficacy at 12 months, with target lesion failure (TLF) occurring in 4.2% of patients, with only 1 event of scaffold thrombosis.^21^ Despite these initial successes, the company has been beset by financial difficulties. In early 2019 it voluntarily suspended trading,^22^ and filed for bankruptcy protection in early 2020.^23^

### Magnesium-based BRS

Magnesium in its pure elemental form does not have the radial strength required to prevent acute elastic recoil.^6^ When combined with zinc and manganese, however, the mechanical properties are comparable to stainless steel stents, with low elastic recoil (less than 8%), minimal shortening after inflation (less than 5%), and high collapse pressures (0.8 to 1.5 bar).^24^

Once deployed and in the body, the magnesium gradually breaks down into inorganic ions and is replaced by amorphous hydroxyapatite, a calcium-phosphorus compound. Additional processes, such as electropolishing of the alloy, can slow the degradation process, with complete degradation occurring by 12 months.^25^ Anti-neoproliferative agents are incorporated into an outer layer of PLLA to allow controlled drug elution. Interestingly, *ex vivo* models have demonstrated that the ionic properties of magnesium may have intrinsic antithrombotic effects, driven by decreased inflammatory cell and platelet deposition.^26^

#### The Magmaris program

The Magmaris program began with the AMS 1 stent (Biotronik AG, Bülach, Switzerland), which was bulky, hard to deliver, and limited by significant vessel recoil due to poor radial strength. This led to unacceptably high rates of TLR (45%) and major adverse cardiovascular events (26.7%) as demonstrated in the PROGRESS-AMS study.^24^ The AMS 2 and AMS 3 stents incorporated changes in the strut design, the magnesium alloy, and the outer polymer matrix, aimed at improving neointimal hyperplasia and vessel recoil. The best-performing of these early BRS—namely the AMS 3—was renamed Drug-Eluting AMS 1.0 (DREAMS),^27^ leading to the first-in-man BIOSOLVE-I^28^ clinical trial.

The BIOSOLVE-I trial demonstrated substantial improvements compared to the PROGRESS-AMS study, with TLR rates of 4.7% and TLF rates of 7% at 12 months, but still underperformed in comparison with contemporary stents.^29^ With further improvements in design—such as the incorporation of tantalum markers to enhance visualization, switching from a poly-D-lactc acid (PDLA) to a PLLA outer coating, and improved deployment technique—the DREAMS 2G scaffold was tested in the BIOSOLVE-II and BIOSOLVE-III trials.^30^ Both trials enrolled stable patients with simple *de novo* lesions. A recently presented pooled analysis of BIOSOLVE-II and BIOSOLVE-III demonstrated similar rates of TLF (6.4%, *n*=174) and clinically driven revascularization (3.7%, *n*=174) at 36 months’ follow-up when compared to second-generation drug-eluting stents (DES). No stent thrombosis events were reported.^31^ The ongoing BIOSOLVE-IV all-comers registry, with more than 1,000 patients enrolled, shows similar TLF rates to those of the earlier, smaller-scale BIOSOLVE-II and BIOSOLVE-III trials.^32^

Despite the DREAMS 2G (marketed as Magmaris) scaffold gaining CE mark approval in 2016, the lessons from the failure of the ABSORB program were at the forefront of operator’s experiences with BRS technology. Urging caution, a consensus paper by experts in the field recommended restricting the use of Magmaris in certain areas until further data became available, specifically recommending against the use of Magmaris in situations such as ST elevation myocardial infarction (STEMI), calcified lesions, poor medication compliance, or ostial lesions, restricting its use to stable patients with simple *de novo* lesions.^33^

The third generation of Magmaris, 3G, is ready to start clinical trials. This 3G platform utilizes Biomag as scaffold material, has thinner struts, markers enhancing visualization, and a large matrix of sizes and lengths to allow proper device selection.

### Iron-based BRS

Iron-based devices offer the advantage of being highly biocompatible with high radial strength but have been limited by a long corrosion period and clearance from the vessel.^34^ Previous *in vitro* studies have shown that a 26-mm long, pure iron-based stent releases 41 mg/month of iron into the bloodstream, equivalent to the typical oral intake of dietary iron over the same period.^35^ Animal models up to 18 months have not shown evidence of iron toxicity.^36^ Pre-clinical porcine models have shown that the iron bioresorbable coronary scaffold (IBS) from Lifetech Scientific (Shenzhen, Guangdong, China) displays similar efficacy and safety profiles to current-generation everolimus DES,^37^ but no current data in humans are available.

## FUTURE DIRECTIONS OF BRS TECHNOLOGY

### Scaffold Design

Strut thickness is one of the principal features thought to be a mechanism behind the rates of stent thrombosis seen with early-generation BRS.^38^ Thicker struts (greater than 150 μm) were required to provide enough radial strength to prevent vessel recoil but led to longer resorption times. Additional concerns, such as polymer dismantling and scaffold discontinuity, have also been shown to be factors related to thicker strut designs leading to adverse events.^39^

Future generations of BRS are in development, and, through innovations in stent design, reductions in strut thickness have been achieved (MeRes100, Meril Life Science, Gujaret, India; Mirage, Manli Cardiology, Singapore; MAGNITUDE, Amaranth Medical, Mountain View, CA, USA; Firesorb, MicroPort, Shanghai, China). In addition to allowing smaller crossing profiles, which improves deliverability, thinner struts have been shown to reduce the shear stress at the vessel wall, allowing for less turbulent blood flow, improved endothelization, and reduced thrombus formation.^40^ Thinner struts have also been shown to reduce restenosis and periprocedural myocardial infarction rates.^41^,^42^ Through these innovations in stent and strut design, lower-profile scaffolds have been made possible without sacrificing radial strength. Trials are ongoing to evaluate whether these innovations will lead to improved outcomes compared to early-generation devices.

### Procedural Considerations

Despite improvements in scaffold design, the importance of correct deployment technique cannot be underestimated. Underexpansion and malapposition were the two most common procedural-related factors leading to adverse outcomes in early clinical trials.^43^ With the implementation of the PSP technique steps to mitigate underexpansion and malapposition, the incidence of stent thrombosis was significantly reduced and was comparable with everolimus DES.^44^,^45^ The 4P technique (patient selection, proper sizing, pre-dilation, and post-dilation) is similar and is aimed at preventing underexpansion and malapposition when using MRS. Future trials utilizing BRS should only proceed with such mandatory procedural steps to mitigate these risks and ensure a favorable result.

### Patient and Lesion Selection

The ABSORB and Magmaris clinical programs have also provided several important lessons on patient and lesion selection. Specific anatomic characteristics seem more amenable to BRS with the current generation of scaffolds.

Vessel size is a key factor, with small vessels (<2.25 mm) displaying higher rates of scaffold thrombosis and stent thrombosis,^45^ and large vessels (>3.75 mm) risking underexpansion or scaffold fracture.^6^ Other complex anatomical subsets such as ostial lesions,^46^ bifurcation, severely calcified lesions,^47^ and in-stent restenosis^48^ have all shown inferior outcomes in small-scale substudies and clinical trials.

Patient-related factors and the clinical presentations of patients also play a role. When used in STEMI, both magnesium- and lactate-based scaffolds have demonstrated inferior event rates compared with everolimus DES.^49^,^50^ When used in non-STEMI patients, the Magmaris BRS appears safe when compared to EES at 12 months, but long-term data are not yet available.^51^

Future clinical trials should aim to adequately assess the safety and efficacy of BRS in higher-risk anatomic and patient subsets. With improvements in scaffold design, preservation of vessel lumen and vasomotion may become possible in situations such as in-stent restenosis and chronic total occlusions.

### Dual Antiplatelet Therapy after BRS

Understanding the BRS resorption period is an important factor when considering dual antiplatelet therapy (DAPT) duration. During resorption, scaffold discontinuity and polymer breakdown may provide a nidus of thrombus formation, so DAPT should be maintained until complete scaffold resorption is achieved. As with all DAPT, however, this needs to be balanced against the increased risk of bleeding events. As scaffold technology advances and quicker resorption times are achieved, thus reducing DAPT duration, BRS may become more suitable in patients at high risk of bleeding, but trials will be needed. Current European guidelines treat polymeric and metallic resorbable scaffolds as a single class and recommend a minimum of 12 months of DAPT (class IIA C).^52^

### Future Directions

With the latest improvements of the permanent metallic scaffolds, and their performance with short DAPT without stent thrombosis, the question arises as to what the role of bioresorbable scaffolds should be in 2020 and beyond. Recent data suggest an ongoing TLR rate of 2% per year with second-generation DES.^53^ The BRS technology may provide a way to avoid these late events, but the technology needs to at least perform as well as permanent metallic stents in the short term and better in the long term. This was not achieved with the first generation of BRS technology, and continued iterations of the technology are warranted to meet this goal.

## CONCLUSIONS

The BRS technology still holds promise. The lessons learned from the ABSORB program about patient and lesion selection, deployment technique, and the need for long-term follow-up were all valuable. The key to ensuring that the clinical community does not lose faith and prematurely turn its back on future BRS technologies lies in rigorous, adequately powered clinical trials with long-term (>5 years) follow-up. Ongoing device development focusing on new materials and thinner struts may still allow a path to the “leave nothing behind” strategy.

## Abbreviations

BRS: bioresorbable scaffolds
CoCr: cobalt chromium
DAPT: dual antiplatelet therapy
DAT: desaminotyrosine polycarbonate
DES: drug-eluting stent
EES: everolimus-eluting stent
MRS: metallic resorbable scaffolds
PDLLA: poly-D, L-lactic acid
PLLA: poly-L-lactic acid
STEMI: ST elevation myocardial infarction
TLF: target lesion failure
TLR: target lesion revascularization
